# Supplementary material for: The GA4GH Variation Representation Specification: A computational framework for variation representation and federated identification
Source: Cell Genom. 2021 Nov 10;1(2):100027. doi: 10.1016/j.xgen.2021.100027 (PMC8929418; doi:10.1016/j.xgen.2021.100027)
Supplement: Document S1. Figures S1 and S2 and Tables S1 and S2 [file mmc1.pdf]

Cell Genomics, Volume *1*

Supplemental information

The GA4GH Variation Representation Specification: A computational framework for variation representation and federated identification

Alex H. Wagner, Lawrence Babb, Gil Alterovitz, Michael Baudis, Matthew Brush, Daniel L. Cameron, Melissa Cline, Malachi Griffith, Obi L. Griffith, Sarah E. Hunt, David Kreda, Jennifer M. Lee, Stephanie Li, Javier Lopez, Eric Moyer, Tristan Nelson, Ronak Y. Patel, Kevin Riehle, Peter N. Robinson, Shawn Rynearson, Helen Schuilenburg, Kirill Tsukanov, Brian Walsh, Melissa Konopko, Heidi L. Rehm, Andrew D. Yates, Robert R. Freimuth, and Reece K. Hart

Supplemental Figures

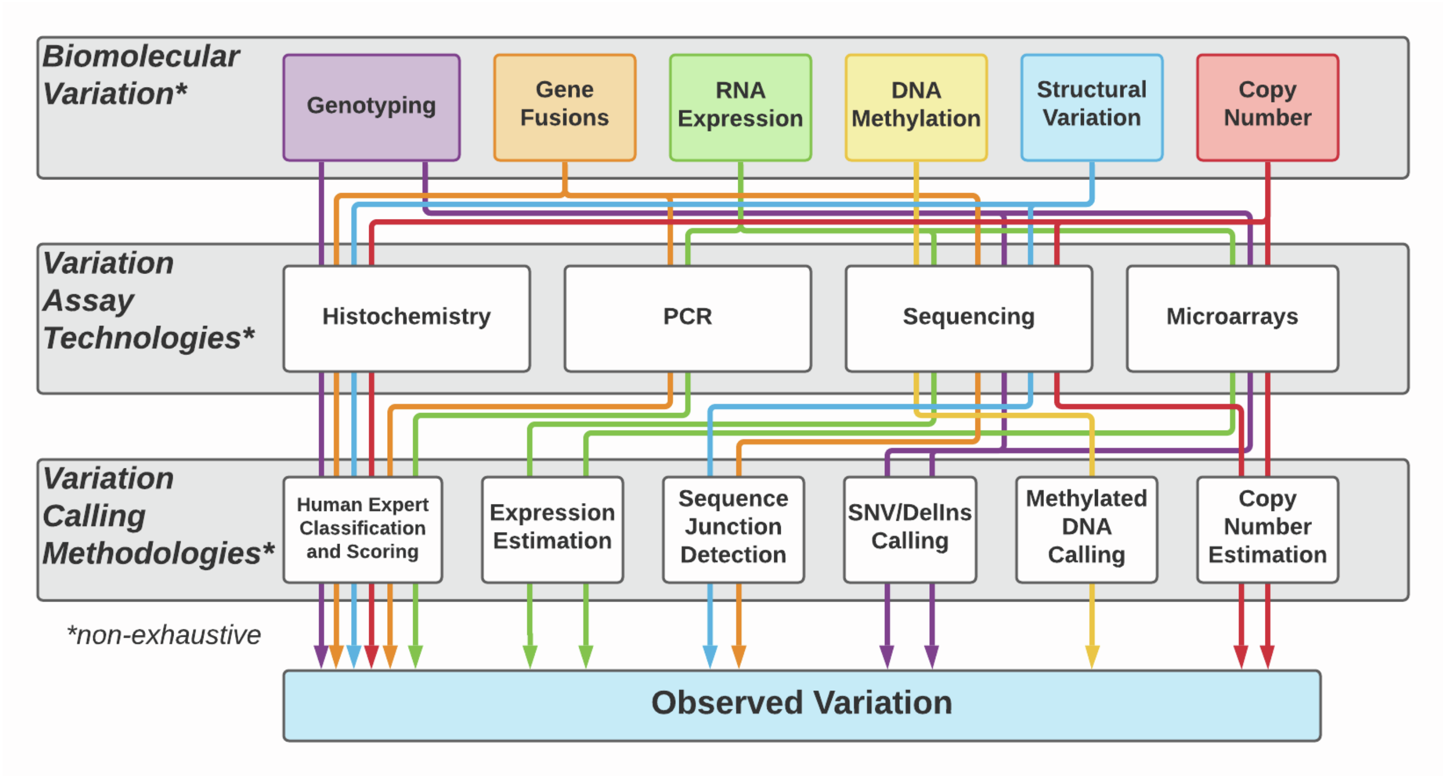

Supplemental Figure 1 - Variation Pathways
A non-exhaustive set of biomolecular variation concepts (**first row**) that are implemented or planned for the Variation Representation Specification. These variation concepts may be assayed (**second row**) and the assay signals evaluated (**third row**) to generate observed ("called") variation for downstream evaluation. Observed variation is compared to knowledgebases linking putative biomarkers to evidence informing clinical decision making. The many pathways (**colored arrows**) from variation concept through assay and evaluation result in a disparate collection of variation representations.

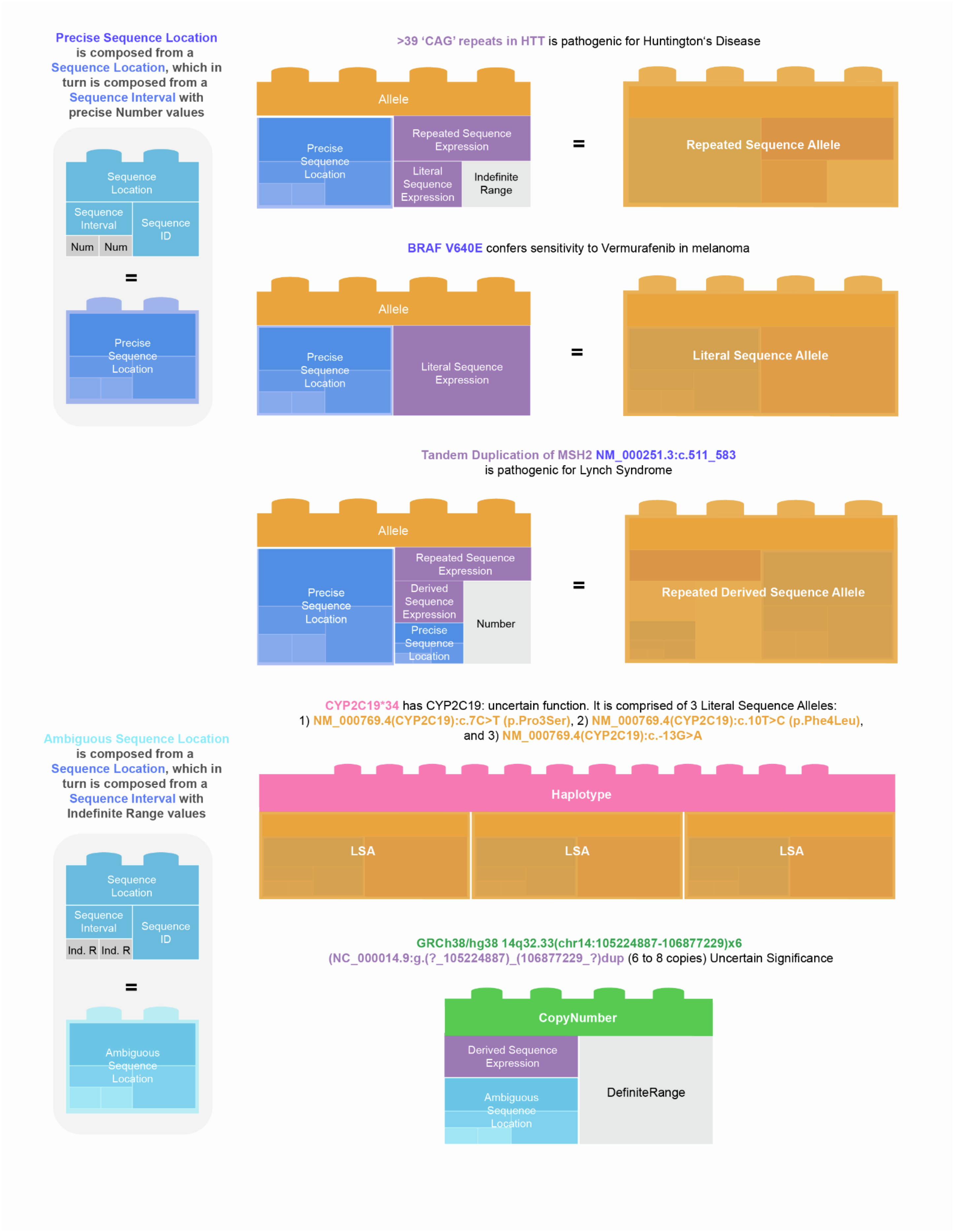
Precise Sequence Location is composed from a Sequence Location, which in turn is composed from a Sequence Interval with precise Number values
Sequence Location
Sequence Interval
Sequence ID
Num
Num
=
Precise Sequence Location
>39 'CAG' repeats in HTT is pathogenic for Huntington's Disease
Allele
Precise Sequence Location
Repeated Sequence Expression
Literal Sequence Expression
Indefinite Range
=
Repeated Sequence Allele
BRAF V640E confers sensitivity to Vermurafenib in melanoma
Allele
Precise Sequence Location
Literal Sequence Expression
=
Literal Sequence Allele
Tandem Duplication of MSH2 NM_000251.3:c.511_583
is pathogenic for Lynch Syndrome
Allele
Precise Sequence Location
Repeated Sequence Expression
Derived Sequence Expression
Precise Sequence Location
Number
=
Repeated Derived Sequence Allele
CYP2C19*34 has CYP2C19: uncertain function. It is comprised of 3 Literal Sequence Alleles:
1) NM_000769.4(CYP2C19):c.7C>T (p.Pro3Ser), 2) NM_000769.4(CYP2C19):c.10T>C (p.Phe4Leu),
and 3) NM_000769.4(CYP2C19):c.-13G>A
Haplotype
LSA
LSA
LSA
Ambiguous Sequence Location is composed from a Sequence Location, which in turn is composed from a Sequence Interval with Indefinite Range values
Sequence Location
Sequence Interval
Sequence ID
Ind. R
Ind. R
=
Ambiguous Sequence Location
GRCh38/hg38 14q32.33(chr14:105224887-106877229)x6
(NC_000014.9:g.(?_105224887)_(106877229_?)dup (6 to 8 copies) Uncertain Significance
CopyNumber
Derived Sequence Expression
Ambiguous Sequence Location
DefiniteRange

Supplemental Figure 2 - Building Blocks of the Variation Representation Specification

Components of the specification can be used in many different constructs. Sequence Locations can be defined once and added as a component by reference in larger constructs. Haplotypes, for example, are composed from Alleles. Shown here, a Haplotype (pink brick) is constructed of three Literal Sequence Alleles (LSA; orange bricks). Literal Sequence Alleles are in turn composed from Literal Sequence Expressions (purple bricks) and Precise Sequence Locations (blue bricks) or Ambiguous Sequence Locations (light blue bricks), which in turn are composed from Intervals, Sequence IDs, and supporting primitive concepts. These same underlying components are used to support other VRS variation types, such as Copy Number (green bricks).

Supplemental Table 1 - Features of Variation Representation Specifications

Specification	Purpose	Reference Types	Sequence Coordinates	Allele Normalization	Reference
HGVS	Human readable variant descriptions	Sequences	Residue	3-prime shifted	http://varnomen.hgvs.org/
ISCN	Human readable cytogenomic events	Cytobands	N/A		https://www.karger.com/Book/Home/271658
SPDI	Human readable variant descriptors	Sequences	Inter-residue	Full-justification	https://www.ncbi.nlm.nih.gov/variation/notation/
PGx	Human readable CYP haplotype descriptors	CYP Alleles	N/A		https://www.pharmvar.org/criteria
VCF	Flat-file variant records	Chromosomal Sequences	Residue	Left-shifted	https://samtools.github.io/hts-specs/VCFv4.3.pdf
MAF	Multisample flat-file variant records	Chromosomal Sequences	Residue	Left-shifted	https://docs.gdc.cancer.gov/Data/File_Formats/MAF_Format/
VRS	Inter-system variation exchange	Sequences, Cytobands, Genes	Inter-residue	Full-justification	https://vrs.ga4gh.org/

Supplemental Table 2 - VRS Implementations

Resource	Usage	Language	Status	Example / documentation
BRCA Exchange	Allele search	Python	Live	https://brcaexchange.org/backend/data/vrid/?vr_id=ga4gh:VA.Z6PI2AKspOXLbCgmyw6JYQxRAoF_gi92
ClinGen Allele Registry	Allele translator	C++	Live	https://reg.clinicalgenome.org/vrAllele?hgvs=NC_000007.14:g.55181320A>T
ELIXIR Beacon Network	Variation search	Python	In progress	
NCBI	Allele id constructor (POST)	C++	Live	https://api.ncbi.nlm.nih.gov/variation/v0
VICC MetaKB	Variation search	Python	Live	https://search.cancervariants.org/api/v1/associations?size=10&from=1&q=ga4gh:VA.mJbjSsW541oOsOtBoX36Mppr6hMjbjFr
VICC Variation Normalizer	String parsing to VRS	Python	Live	https://normalize.cancervariants.org/variation
vrs-python	Open Python Implementation	Python	Released on PyPI	https://pypi.org/project/ga4gh.vr/
AnyVar	Variant Registration Service	Python	In progress	
MyVariant.info	Variation search	Python	Planned	
Ensembl	Variant Recoder and VEP	Perl	In progress	
